# Exploring Pictorial Health Education Tools for Long-Term Home Care: A Qualitative Perspective

**DOI:** 10.3390/healthcare8030205

**Published:** 2020-07-09

**Authors:** Fang-Suey Lin, Hong-Chun Shi, Kwo-Ting Fang

**Affiliations:** 1 Graduate School of Design, National Yunlin University of Science and Technology, Yunlin 64002, Taiwan; linfs@yuntech.edu.tw; 2 Department of Information Management, National Yunlin University of Science and Technology, Douliu 64002, Taiwan; fangkt@yuntech.edu.tw

**Keywords:** pictorial health education tools, long-term home care, nurses and home caregivers, communication, case study, a qualitative perspective

## Abstract

Regarding long-term home care needs, nurses need to communicate effectively and reasonably when teaching home caregivers. Designers can assist medical staff and develop pictorial tools to enhance communication. The purpose of this study is to explore a theoretical basis from the perspective of designers, patients’ home caregivers, and medical staff to construct a theoretical framework that can jointly develop pictorial health education tools and healthcare system. The qualitative methods, including in-depth interview and observation, are applied to this study; ground theory sets out to construct a framework from the verbatim transcript of the interviews. Based on interview results, six axial codes were extracted: (1) the method of interdisciplinary cooperation; (2) medical research ethics; (3) communication methods; (4) forms of health education tools; (5) development of health education tools; (6) home care intubation procedure. Eight groups of home caregivers offered suggestions from their experiences. The designers need to assist medical staff to solve real problems, pay attention to professional norms, and forms of cooperation. Health education tools need to meet the needs of medical staff and home caregivers and designers should pay attention to the processes of communication. This study can also assist in interdisciplinary cooperation to explore the theoretical basis of pictorial health education tools for nurses in the context of long-term care at home.

## 1. Introduction

Long-term home care is normally provided by non-professional medical staff. Nurses need to provide the necessary help to long-term home caregivers and patients, but cooperation and communication between the two can become a problem. As the elderly now live longer, the demand for improved services in long-term home care is growing [1] and, as a result, there are increasing demands on nurses’ resources. With interdisciplinary collaboration needed in home care settings, designers can intervene to improve the home care system, to offer different solutions according to different home care needs, and to solve concrete operational problems [2].

To improve the service quality of long-term home care, many researchers introduced health education tools in the field of health care. This included pictorial tools for interventions, to help those with low literacy levels enhance their understanding of health knowledge [3] and improve the recall process of patients after treatment [4]. Furthermore, using images in an environment can reduce negative emotions [5]. In cooperation with designers, pictorial health education tools can be developed for long-term home care to facilitate the operation of medical staff and help patients and caregivers understand professional terminology. As a strategy to cultivate creativity, interdisciplinary cooperation is a form of teamwork to develop knowledge that can promote the integration of different fields of healthcare, and has an important impact on the overall development of learners [6]. There may be obstacles to interdisciplinary cooperation in a hospital, more so for nurses than for doctors [7].

Ageing is a relatively serious concern in the region where the researchers are located. Many elderly people need long-term home care and most of their children employ home caregivers. Most caregivers are foreigners with poor language skills and new caregivers lack relevant experience in long-term home care. Researchers have long paid attention to the skills of foreign caregivers and found that, as well as language problems and lack of nursing knowledge, they are afraid or unwilling to communicate with nurses, so mistakes may be made in the home. Based on preconceived experiences and literature, the researchers thought that pictorial health education tools could assist nurses or home caregivers. The purpose of this study is to explore a theoretical framework from the perspective of designers, home caregivers, and medical staff, and jointly develop pictorial health education tools to enhance communication and cooperation between medical and non-medical staff in the home care setting. Therefore, in this study, it is necessary to examine the following aspects in the qualitative research: (1) what is the perspective of medical staff on the use of pictorial health education tools; (2) what is the perspective of caregivers when using pictorial health education tools; (3) designers’ focus on interdisciplinary cooperation in developing pictorial health education tools.

## 2. Literature Review

### 2.1. Health Literacy of Long-Term Home Caregivers

Home caregivers need to understand the expertise, procedures, and practices in the field of nursing. Knowledge of long-term home care is rooted in the health literacy of home caregivers and the application of knowledge comes from “individuals, organisations, environment and knowledge itself” [8]. Health literacy enables the public to acquire knowledge, understanding, skills, and confidence in relevant health information, which is a positive factor in nursing and needs to be considered throughout the social care system [9]. Health literacy is also defined as the relationship between patients, caregivers, and health professionals in the health care system. Health literacy involves skills that can be taught. During recent years, the relationship of nursing transformed from leader-centred to patient-centred, which changes the structure of the medical system and thus, improves patient safety and satisfaction [10]. Health literacy should be concerned, not only with operational skills, but also with changes in health behaviour, involving the development of multiple domains [11]. Health education tools to improve health literacy are not limited to development and use but should also focus on generality so that medical staff in the process of improving the health literacy of stakeholders in the family environment is similar to the development of nursing teaching tools that are suited to the medical environment [12].

Long-term caregivers only take basic training lessons and lack of medical professional background, but they play a very important role in home care. Medical terminology often confuses patients and the use of relevant strategies in nursing can reduce its negative influence on patients during a conversation. The popularity of medical vocabulary promotes understanding, enables patients to better socialise in the medical environment, considers the background of the patient’s environment, explains terminology, and enables doctors to pay attention to the expressions and reflections of patients when communicating [13]. When patients think the technical terminology used in clinical diagnosis is difficult to understand, reading and understanding written text is very important. More specific information and vocabulary are needed to make it easier for patients to understand, and adding pictures to a verbal description can improve patients’ comprehension. In the medical training process, the patient’s ability should be taken into account, as sometimes it is better to use pictures to illustrate the information to make it easier to understand and easy to apply at home without medical staff [14].

### 2.2. Pictorial Health Education Tools

Medical health education tools are morphologically diverse. New technology is used to place graphic content in electronic equipment; digital technology combined with images can improve learning fluency, reduce the cognitive gap, and improve doctor–patient communication [15]. Compared with text, the images have an important influence on the effective communication of information, which can improve concentration, memory, and understanding. Images can also change the public’s attitude to health education, especially for those with literacy and reading difficulties, and encourage those more willing to follow the content of image information practices [16]. The use of pictorial health education tools in medical institutions, nursing institutions, and long-term home care environments can help caregivers understand the instructions from medical staff, enhance the nursing process, and improve caregivers’ health literacy and communication skills [3]. Some studies used experimental approaches to assess the influence of pictograms on people’s understanding of information about health care. It was proved that when the text was accompanied by a picture, it enhanced people’s recall of the text, and suggested that graphic designers and stakeholders can participate in the study and that use the reasonable way to evaluate pictorial use [17]. Pictures are also used in language training, assisting low or no speakers to acquire language skills. The use of images will help communication, making it easier to understand and remember [18]. From the perspective of auxiliary functions, for students who lack knowledge, pictures are the best way to enhance learning [19,20]. A picture can make the information interesting if features can be memorised, for example, colour, complexity, and meaning [21].

### 2.3. AAC Is Applied to Doctor-Patient Communication

Augmentative and Alternative Communication (AAC) along with the progress and development of technology, provide innovative solution for a wide range of users with various language barriers [22]. With technological development, AAC was more widely applied to nonverbal communication, clinical communication, and language. Pictures are an important part of AAC as it expands its application further and the conversion from language to an image requires detailed evaluation [23]. AAC’s core vocabulary is suitable for improving children’s oral expression and has been proved effective [24]. AAC has been used in long-term care institutions to improve the quality of care for the elderly [25]. It has also been applied in the context of intensive care unit and diversity of auxiliary communication tools. However, medical staff have little understanding of the communication board and the communication strategies used by medical staff may affect the communication ability of patients [26]. According to different needs, AAC uses high-tech, low-tech, and non-tech assistance. The emotions of stakeholders should be taken into account, which will affect their willingness to communicate [27] and the use of AAC for emotional communication between patients and caregivers can improve the patients’ quality of life [28].

### 2.4. Grounded Theory

Grounded theory is applied by researchers who want to solve a problem in a specific environment; it provides a theoretical basis before the research is formally started. Researchers identified pre-understanding information is correct and operable in the process of practice, and in the process of concrete operation, grounded theory based on the interviews to correct the research content, according to the methods of observation, proving the problems of the research. Grounded theory is the earliest research applied in the field of nursing by Glaser and Strauss. They defined grounded theory and believed that it could be used as the basis for better theoretical construction in social research [29], to develop theories in data research and show the context of data [30]. The construction of theory needs to constantly examine the correctness of theory and the perfect nature of data, fully explain the phenomenon, and add the original theory to the new concept [31]. However, the data collection methods of grounded theory have been controversial. Researchers found that the data are objective after the data are analysed and coded by researchers to form a new framework. Glaser and Strauss thought that researchers anticipated the objective, made assumptions about scope and conditions, and interpreted and constructed study phenomenon [32]. The cooperation of people with different research backgrounds makes grounded theory a scientific qualitative research method. It holds the tradition of symbolic interaction theory and emphasises daily life experiences and self-cognition of basic parties [33].

## 3. Materials and Methods

### 3.1. Study design and Structure

**Figure 1:**
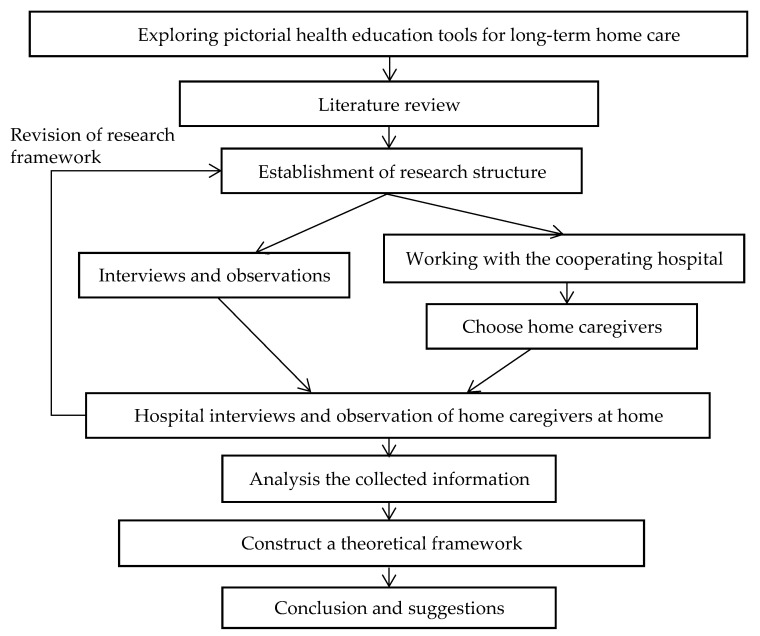
Exploring pictorial health education tools for long-term home care research.

The researchers collected information about home healthcare, tracked the theme of the pictorials used by healthcare assistants, and conducted interviews and observation surveys to collect the information from the perspective of designers, patients’ home caregivers, and medical staff to construct a theoretical framework, and then compared this with existing communication theories and literature. A construction framework was established that enabled the designer to attempt to develop pictorial health education tools for long-term home care (Figure 1).

### 3.2. In-Depth Interview and Observation Method

The researchers initially proposed to develop tools for intubation procedures, but the nurses recommend another alternative project because these procedures are performed by professional nurses and not by home caregivers. However, home caregivers could be trained in basic nursing and daily cleaning; they could also understand the routine intubation procedure, recognise if patients were in discomfort afterwards, and report this in a timely fashion to the professional nurses.

In the areas surveyed, there was a large proportion of foreign home caregivers. Long-term home care hospital nurses are responsible for training foreign home caregivers, but when there is poor verbal communication, it can lead to a decrease in overall communication and influence the learning efficiency and understanding of nursing operations. The problems faced by the researchers in determining the research content became clearer after a discussion on what images to use as communication tools in the process of teaching, how to help home caregivers, especially foreigners, with the intubation of patients, and the feasibility of developing apps for medical aid.

At the beginning of the study, interviews were conducted with hospital staffs about home caregivers’ learning experiences and difficulties. These were two times semi-structured focus group interviews in the hospital. The interviewee had to consent to the recording and the interviews were adjusted according to their personal experience. A manuscript was developed to help with the questioning (Table 1). Respectively, from what the interdisciplinary cooperation norms were [6], the manner of nurses’ communication [34], problems in home care procedures [35], expectations for developing health education tools [36], and nursing care for intubated patients [37]. In exploring the theoretical model, we only interviewed those working in hospitals, and the home caregivers were observed and recorded to confirm their intention of using pictorial health education tools.

### 3.3. Participants

The participants interviewed and observed in this study were divided into three groups, designers, medical staff, and home caregivers. In previous research, a good relationship of cooperation and participation with the hospital was established. Hospital managers were willing to assist the participants in the groups to solve specific problems and tasks. Participants described their ideas and perspectives during the team meeting. One group included five hospital participants: the hospital director, nursing supervisor, and nurses. The other group had six design participants in design research and practice, including a teacher at the school’s research institute and five assistant researchers. These participants conducted a preliminary group interview to build the theoretical framework and they also participated in later research practice. Nurses from the cooperation hospital provided eight groups of cases, all of which were home caregivers of intubation patients. Operational observation and random interviews were conducted with the home caregivers when the theoretical framework of this study was established.

### 3.4. Content Analysis

According to interviews with the medical staff, three researchers with design backgrounds were employed to code together. The three researchers had experience in design strategy, teaching, and image design, respectively. From the perspective of the designer, the interview results with the medical staff were analysed and common words confirmed by trigonometric verification. The purpose was to reach agreement among the designers with different backgrounds to find out feasible reference suggestions and form a theoretical framework when using pictorial design to solve specific problems. The interview text was repeatedly read and confirmed. Combined with the data processing method of grounded theory, researchers conducted open coding to form a common coding book. Meanwhile, the operation method and formula of content analysis [38] were used to increase the credibility of the selection of each sentence during the interviews. This formula was used to check the degree of agreement between the interview verbatim manuscripts and the reliability of interview manuscripts. The degree of mutual agreement required the joint participation of the three coders to screen common statements and then, according to the degree of mutual agreement, encode the reliability of the three coders.

The degree of mutual agreement is as follows:

(1) $$\mathrm{Degree}\;\mathrm{of}\;\mathrm{mutual}\;\mathrm{agreement}\;=\frac{N\times M}{N_{1}+N_{2}+\cdots Nn}$$

(*M* is the number of the fully agreed sentences, *N*_1_~*N_n_* are the number of participating researchers).

The content analysis reliability calculation is as follows:

(2) $$\mathrm{Reliability}=\frac{n\times (\mathrm{Average}\;\mathrm{mutual}\;\mathrm{agreement})}{1+\left[\left(n-1\right)\times \mathrm{Average}\;\mathrm{mutual}\;\mathrm{agreement}\right]}$$

### 3.5. Research Ethics and Limitations

This study passed the National Cheng Kung University Human Research Ethics Review Committee review. The home caregivers who participated in the research signed the informed consent and agreed to be observed and interviewed. The research process was conducted with the nurse supervisor in the hospital.

This study adopts the qualitative research method and has specific research objectives for foreign caregivers who deal with home care intubation. It is only limited by the theoretical reference for developing pictorial health education tools. Due to geographical limitations, this study may not apply to other situations with home care needs as other areas may require more diversified forms of graphical tools, solicit the advice of medical staff according to different studies, and require users to have more reasonable and scientific evaluation forms.

## 4. Results

### 4.1. Open Coding and Axial Coding

This study conducted two in-depth interviews lasting about 60 min, which were sorted into verbatim drafts and coded. Based on the empty framework, open coding was conducted by the three researchers without any discussion with each other. In the first calculation of reliability, the number of keywords in the verbatim text of the interviews was 20, 26, and 21, respectively; there were too few common keywords because the reliability value was less than 0.6, so it was rejected. Before the second reliability analysis, the researchers held a meeting to analyse this and found that, in the first coding process, the reason is that the coders focused more on the researcher’s problem rather than the nurse’s recommendations. In the second coding process, the coders were required to find the nurses’ viewpoints on the operational needs of teaching home caregivers from the perspective, the designer based on the interview results and to solve the problems of home care with the help of the design method, before coding again. After discussion, the three coders extracted the number of keywords, which were 23, 22, and 22, respectively and there were 20 common keywords. This result is shown in Table 2. The common statement calculation formula of the final triangulation test was 20 × 3 / (23 + 22 + 22) = 0.896 and the reliability value was higher than 0.6.

According to the interview results (Table 3), the interviewees of different professional respondents are different. Based on previous experience, the hospital management believed that the graphical representation should be the hospital’s design of the guidance system, rather than clinical operation. While researchers raised the issue of home care, hospital administrators raised the issue of operational ethics. Therefore, when designers conduct research cooperation, according to the groups involved in the research problem, they should pay attention to the physical condition of stakeholders and the degree of physical contact in the process of research and development and abide by ethical norms. Nurses were very concerned about the operating details and thought they might occur problems in communication during the nursing process. The nurse and patient communication tools, for example, used phonetic or pinyin boards. More patients were spelling slowly, reducing communication efficiency. The researchers mentioned that in long-term home care, communication with the elderly being intubated, if there is a graphical representation, it will make it easier for home caregivers to do it.

According to the open coding process discovered that hospital managers were concerned about the interdisciplinary cooperation method and ethical medical research, and thought that the researchers should have empathy, follow nurses in clinical operation and adjust the research direction according to the requirements of the nurses. Furthermore, research information should be as detailed as possible and research ethics in the process of cooperation was very important, especially in gaining consent from the family. If the patient or family did not give consent, the research could not go ahead.

Nurses had some doubts about whether design can actually help understanding the caring process of patients with intubation operation, they asked researchers to develop a sample of graphic tools based the videos and written materials in patients with intubation operation. This also avoided ethical issues about intubation and enabled the realisation that the placement of the tube could be difficult for non-specialists. When the researchers mentioned health education tools for learning, nurses were more inclined to use traditional textbooks. This may be associated with aseptic manipulation in the operational process as there may not be anywhere to put up wall charts in the home environment or use electronic devices, such as a mobile phone, might not be able to solve the problem of hands operation, other wearable device size is too small, not convenient to prompt operation. Therefore, finally put forward the health education tools A4 paper printed format.

Nurses believe that long-term home care should not focus on intubation but detailed care in the home environment. Although nurses consider these actions to be basic, the layman may have difficulty in the home care process. Studies have shown that nurses believe that more than half of home care infections are caused during intubation [39] and infection or cleaning in the home environment is a very important issue. Work for home caregivers, in addition to the problems of intubation care, involve physical work. When nurses described the communication behaviours of caregivers with years of nursing experience, they indicated that videos were not used for teaching, but direct demonstration was given on the spot. In terms of the number of home caregivers at present, foreign caregivers are in the majority and nurses used paper to communicate with patients. 

In the process of interdisciplinary cooperation, detailed communication was required during the interviews because of the different professional backgrounds involved. Although there are many communication problems between nurses and patients or caregivers, new health education tools had not been considered to improve the current situation. Nurses’ communication, especially with patients who cannot make a sound, is based on guesswork and experienced judgment; communication with foreign caregivers cannot be conducted verbally. Given these two communication issues, burses still think they can make a judgement based on previous experience and this may affect the accuracy of the communication process. From the perspective of developing pictorial health education tools, the researchers with design backgrounds believed they can effectively assist nurses in communicating and teaching; thus, improving the quality of home care operations. However, sometimes these researchers failed to pay attention to detailed operational procedures and ethical issues in the cooperation process. Despite the good intentions of the researchers, nurses were still reluctant to cooperate because their daily work is very complicated and they need to communicate with many people. Information visualisation can promote the basic knowledge of home care for the public and visual health education tools can improve doctor-patient communication and services if professional design skills are used. It is difficult for designers without a nursing background to assist nurses because they are not familiar with the professional operation of nursing. If they do not communicate in-depth with nurses, the design output may not have a practical application in the field. On the other hand, designers also need to learn about nurses’ workflow and form clear steps in developing graphics. During the interviews, the researchers developed six axial coding statements, namely, the method of interdisciplinary cooperation (AC01), medical research ethics (AC02), communication methods (AC03), forms of health education tools (AC04), development of health education tools (AC05), and intubation home care operations (AC06).

### 4.2. The Quantities Relationship between Facts and Axial Coding in Interdisciplinary Cooperation

According to the operational steps of grounded theory, after axial coding was confirmed, the researcher selectively coded according to the context of the interviews. The selective encoding table represented the relationship between axial and interview facts and how they overlapped because it was necessary to find factual descriptions of the mutually supporting relationship between different axials in the context relationship and the specific quantitative relationships used in Table 4.

**Proposition** **1.** *Interaction between the method of interdisciplinary cooperation (AC01) and medical research ethics (AC02).*

● *Researchers can follow them on the front line, the research topic may change, the data will be more refined. (Researcher: We need to come over frequently for our investigation). When doing research, patients are involved and there will be patient privacy, so relevant regulations should be adhered to* (CA01-54).
● *(Researcher: Let’s start with the case today). Learn as much as you can from nurses, observe their problems and help solve them. The design of the icon registration section has been developed and published before* (CA01-58).

**Proposition** **2.** *The method of interdisciplinary cooperation (AC01) influences the intubation home care operation (AC06).*

● *(The design background researcher explained that the ethical review of the study had been done and suggested that the study would not involve patients. In the study, the researcher showed uncertainty about the health education tools in the interdisciplinary cooperation process and asked the nurses to give relevant advice) If you want to take pictures (as the record), you may have to match our(the nurses) time and then you can take pictures beside us when we are working. That step will slowly decompose* (CB02-15).
● *So, let’s start with three-tube care, which is to give you the video and the paperwork. Map out the steps and bring them up when we visit the home. Most of the body cleaning is fine, only the technical aspects, such as turning the patient over and getting them in and out of bed* (CB02-65).

**Proposition** **3.** *Intubation home care operation (AC06) influences medical research ethics (AC02).*

● *(The nurses suggested that the intubation procedure should be photographed and the researcher confirmed if the photography and video were available). Yes, but we need the consent of the patient and the family first* (CB02-18).

**Proposition** **4.** *Communication methods (AC03) and health education tools development (AC05) influence each other and communication methods (AC03) also influence the method of interdisciplinary cooperation (AC01) and form of health education tools (AC04).*

● *(The researchers asked about the origin of the foreign caregivers). Indonesia predominates, as does the Philippines. (The researcher asked about the language of the health education tool, whether a simulation demonstration could be conducted). We can start with a medical dummy. But we need to know the structure of the medical dummy first. Whether the nasogastric tube can be inserted is uncertain* (CB02-37).
● *We could look for a similar standard movie for your reference or we could try to send you a video. It is mainly about our technology of placing tubes and care. (Researcher: We are probably going to bother you if you have to teach them what to prepare beforehand and then you have to do the homework. After all, we are non-professional majors, but we will watch relevant videos first to understand. If there are any questions, we will ask you again. Have you used videos to teach foreign caregivers in the past?) There is no video, just direct on-the-spot demonstrations* (CB02-49).
● *(Researcher: During direct demonstration, in which cycle did the problem appear?) The nurse replied: Communication. (According to the interview, the researcher further proposed drawing a step-by-step diagram after watching the video to see if a wall chart could be adopted. If there was any problem, it could be adjusted). So, let’s start with three-tube care, which is to give you the video and the paperwork. Map out the steps and bring them up when we visit the home* (CB02-57).
● *What if it’s A4 size? Wall charts are not convenient in case there is no place to hang them* (CB02-148).

**Proposition** **5.** *Intubation home care operation (AC06) and medical research ethics (AC02) influence the communication method (AC03).*

● *Most of the body cleaning is fine, only the technical aspects, such as turning the patient over and getting them in and out of bed. (In terms of studying, researchers suggested there was very little data on the operation of basic nursing skills and caregivers would not buy such material. However, home caregivers’ skills are important and improper operation may result in injured patients. Practical demonstration may be easier to understand, the video still has disparity compare with the fact operation, and then the researcher consulted the nurse commonly the method of communication with the intubation patient at present). Written on paper, Chinese characters* (CB02-83).
● *(The researcher asked about preparing the consent form and nurses told them to prepare it by themselves as they needed to know the language used by foreign home caregivers). Indonesia predominates, as does the Philippines* (CB02-26).
● *You can’t read the text directly, but we can use a medical dummy, then provide written information while giving guidance. (Researcher: Are there any examples of how patients cannot communicate? Nurse: I have one here at the nursing home. Researcher: What do you use when you need to communicate with patients?) He (the patient) communicated with me using his hands and feet because he can only say a few words; the patient got angry. The patient can make breathing sounds, but I can probably understand them. In terms of communication, he only listens to what he wants to hear* (CB02-173).

**Proposition** **6.** *The form of health education tools (AC04) influence intubation home care operation (AC06).*

● *(We) think you can use A4 size to make a small manual and then coil it into a book so it’s easy to turn pages. This will be easier than wall charts. (The researcher proposed preparing several forms of health education tools. Only after testing can we know which pictorials can be truly understood. The nurse’s assistance and modification may be needed in the drawing.) In my opinion, intubation should not be the main focus of carers, but care should be mainly about disinfection and how to observe the principle of sterility. It is mainly about the care of the tubes. We (nurses) have to change the tubes* (CB02-156).

### 4.3. Home Caregivers’ Point of View

Through observing home caregivers, we found that their experience was valued by most families, but the initial source of nursing knowledge was diversified through either professional or non-professional channels. Most of the intubation home care was learned only by temporary learning or contact with users. Some caregivers need on-the-job training in nursing homes or are trained by nurses; they can also learn from a family member or mutual learning between home caregivers. Regardless of the stage of training or experience, home caregivers can learn from doctors or nurses, according to each patients’ needs. A long period spent in home care can build a good relationship with patients.

Before intubation caring, nurses would ask the caregivers about their patients’ condition. Some patients used more than one intubation method, but nurses seldom asked their families. Home caregivers undertake detailed observation of their patients in home care. The process of removing nasogastric tubes is very painful for the patient. In addition to the nurse’s verbal soothing process, the caregiver must be beside the patient to comfort them. The patient cannot express his discomfort verbally, only by expression, shouting and gesturing. Although some patients are old, they are still fully conscious and they did not want to be seen by others when the tubes were replaced. Therefore, they were only observed by nurses and main researchers.

In the process of independent home care, home caregivers need to keep close contact with nurses. On the one hand, intubation needs to be performed by nurses, and on the other hand, home caregivers need to provide timely feedback about their patient’s condition. It is easy for caregivers to forget the steps. Sometimes they use mobile chat software to friends or nurses about the caring details.

Home caregiver 2 indicated that the manual was effective and that bilingual text can help them understanding the care process correctly. Due to language limitations, the researcher provided home caregiver one case an intubation home care graphical manual for their reference. She pointed out the illustrated diagram and Indonesian text helped her understand how to care properly. The case of home caregiver 2 will resign her job soon and replace a new caregiver. During the transition period, home caregiver 2 will need to teach the new home caregiver using a graphic manual. In case of home caregiver 3, family members helped with teaching and supervision, indicating that having this graphic manual was helpful for their caregiver’s learning and recall. Along with the graphics, corresponding native language assistance was provided. In the case of home caregiver 4, she did not learn about the procedure for nursing the nasogastric tube; therefore, the nurse had to teach her from the beginning. Home caregiver 5’s patient was elderly, but was still fully conscious and could understand the intubation graphics; she found it very helpful. In the case of home caregiver 7, the caregiver can speak mandarin fluently, and her learning ability quite good; she had taken care of her particular patient for about six months and could understand the manual. The case of home caregiver 8 was quite old, and took care of the patient by herself most of the time; she took quite a long time to learn the material and had a slower recall. Nurses suggested that for new or slow learners, it was helpful for them to learn how to care for patients using graphic health education tools. First, they pointed out the steps to be done with a manual and then demonstrated the actual operation. Use a graphic manual to assist nurses in teaching process communication, and the caregivers can go over the manual by themselves. The graphic is also helpful to recall the operation process of nursing care. The caregivers cannot read Chinese, and graphics with Indonesian keywords can be understood well.

### 4.4. Theoretical Framework for Pictorial Processes in Long-Term Home Care and Communication Problem-Solving Strategies

**Figure 2:**
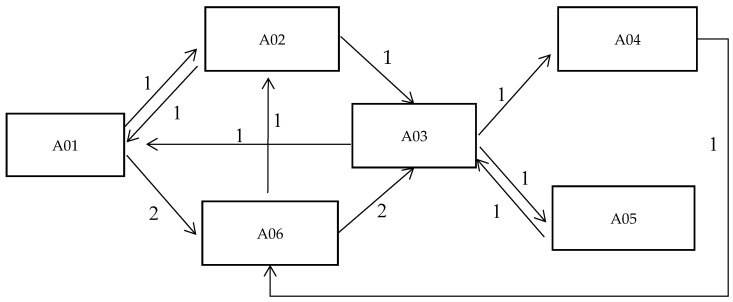
Theoretical framework: Augmentative and Alternative Communication (AAC) diamond model

The researchers put forward the case for developing a health education tool for intubation nursing. They had to respect the wishes of hospital nurses, but also make the tool convenient for home caregivers. After the tool was developed, according to the theoretical framework, it was promoted to nurses and home caregivers to improve communication in teaching and learning. The tool was based on the relationship of selective coding with facts and graphic information was added, according to an adapted AAC diamond model. The diamond model plays an important role in the problem-solving process and is also used in the teaching process of tool development in the health sector [40]. A theoretical framework similar to the diamond model was discovered after the interviews. Combining or replacing oral expression in communication, it discusses the application of the assisted oral method to communicate with home caregivers in the field of medical care and develops a communication tool for health education suitable for application (Figure 2).

Communication in the medical environment is a very special social communication and the relationship between doctor–patient can affect how they interact. Regional studies suggest that many factors can cause poor communication between nurses and patients, such as language ability, nurses’ workload, remuneration, and time management, all of which affect the nurses’ communication behaviours and intentions [41]. Factors such as multiple uses of nursing tools, training, and clinical experience may influence nurses’ willingness to use them [42]. Instrumental communication translates into tool-based teaching and learning relationships. In essence, the assistance tool transforms the communication mode between nurses and caregivers, improves the understanding of the medical process, and indirectly affects the doctor–patient relationship. However, different patients may have different standards of satisfaction, not only the satisfaction in the nursing process but also their expression, movement, tone, and environment. The emergence of communication tools and intermediaries to coordinate and communicate the needs of both sides can help the medical and the patient side to make better medical decisions and provide patient-centred services. Nurses described a home setting to the researchers:

*In the past, if a caregiver had a poor memory, you gave them a video. The average person can’t remember so many steps and precautions. Nurses teach in Indonesian or Vietnamese using health education leaflets provided by existing institutions and as there are no graphics, they can only describe them orally. To see how well caregivers are learning, it would be better to have a pictorial manual.*

## 5. Discussion

### 5.1. Assist Medical Staff to Solve Real Problems

During the interviews, when the researcher proposed the need for frequent communication with nurses and relevant field observations, hospital managers repeatedly stressed that researchers should work with nurses to present real data, that patients’ privacy should be covered by medical research ethics and that researchers should empathise with interdisciplinary research [43]. Because there were many cases of interdisciplinary cooperation, there was a need to develop tools that helped nurses solve real practical problems. There was a concern that cooperative research did not understand the application, the hospital would lose momentum and the research would have to be applicable in practice. Nurses needed the tools to facilitate teaching carers or learning content, but in researching what was needed, the designer had to create the operation details as a beginner. The researchers wanted to choose the relevant intubation content but found that nurses who teach in the home care setting needed to teach nonprofessional carers how to avoid adverse impact on the patients. Therefore, in cooperation with the designers, nurses decided whether to use textbooks or videos and determine the most needed aspects of home care. To demonstrate the intubation procedure, the designer lacked the medical knowledge, especially in the home care setting, making it vital to cooperate with medical staff, when necessary, to understand the problems of home care in the learning process. At the same time, nurses lacked the design skills of drawing flowcharts, which is what designers are good at. However, at times, nurses did not think this part was important because of their workload. Cross-field cooperation is a process where different specialities complement each other and exchange mutual professional cognition, which may ultimately affect the form of intubation operation in a home care setting.

### 5.2. Professional Norms and Forms in Interprofessional Cooperation

Medical personnel have repeatedly emphasised medical ethics and believe that in the process of home care cooperation, consent should be obtained from the patient’s family to avoid unnecessary disputes. It is suggested that researchers should review medical ethics, whether in the process of intubation care or the development of health education tools. Stakeholders’ information should be confidential and used only in the study. The hospital should assign a supervisor to guide the entire study process during the nurse’s operation and record the process. As the home caregiver is of mostly foreign nationality, the development and form of health education tools may be affected and text and image should be considered in the design. When nursing professionals and non-professionals have different levels of knowledge and communication ability, misunderstandings may occur. Interventions and tools can form the basis for effective communication [44]. Nurses believe that the biggest problem in the operation process is communication, so a schematic design of the home care intubation process should be completed and then a health education tool, applicable to the home care setting, should be developed. Nurses provide immediate on-the-spot demonstrations of the intubation process, which is the teach-back method [45]. In this study, nurses thought that the researchers should draw images from the video, which may affect the accuracy of the graphics, because of the cooperation at the beginning may increase in their workload, and it was also possible that could influence the willingness of nurses to cooperate. In the interview, nurses had expectations about the form of health education tools. The researchers proposed two different methods of communication (electronic or traditional wall chart), but the nurses thought the form of the health education tool can be an A4 size with colour printing and a Wire-O binding form, mainly related to the nurses in clinical operation convenience.

### 5.3. The Form of Health Education Tools Has to Meet the Needs of Medical Staff and Home Caregivers

In the process of nursing operations, non-medical professionals have some difficulty in understanding and remembering the details. There is a big difference between the hospital side and patient side in terms of medical knowledge, so hospital staff need to recognise the knowledge level of patients to help them understand the content and they should also consider using hints [46]. When the language involved is not the mother language, it affects various social and cultural relationships. To attract the attention of stakeholders, the nursing team should provide relevant solutions for the communication barriers based on medical language [47]. Additionally, foreign carers’ lack of language ability further affects communication. If the family members do not agree, then new health education tools cannot be applied in the nursing process, which will continue to affect communication. Medical personnel and the design professionals were consistent in understanding research ethics and agreed that medical ethics needed to be strictly followed for the process of interdisciplinary research and cooperation to run smoothly.

Nurses thought it was more convenient to make health education tools into a manual. The intubation method was not the main concern of home care; the main focus was to follow the principles of disinfection and asepsis. The medical environment is composed of different services and professionals and effective professional collaboration is necessary to ensure the quality of care for patients and the overall medical care system [48]. The nurses’ opinion on the form of health education tools was different from the designers. The designer hoped to use a more novel technical solution to help the clinical teaching process of nursing, but nurses thought that traditional printed matter was more conducive to the teaching process. At first, the design professionals wanted to draw the intubation teaching steps, but the nurses explained that professionals were still needed in the home care setting to perform the operation as home caregivers lacked the medical knowledge. They stated that home caregivers needed to concentrate on cleaning and hygiene. In terms of home care, the public wants medical institutions to provide flexible services and ensure communication is effective between nurses, home caregivers, patients and their families and that nursing is provided according to the requirements of individual patients, providing humanist care services [49].

### 5.4. Pay Attention to Communication Behaviour in the Nursing Process

Gerber (2016) proposed solving doctor–patient communication problems with philosophical methods. Non-traditional communication methods were applied and new tools developed that used reasonable processes in specific situations that could overcome communication barriers in diagnosis and treatment [50]. In communication theory, Habermas outlined four aspects that included tools, strategies, language and communication. Tools conveyed an object; a strategy was adopted by both sides in reaching a consensus of how to communicate, especially when there was a language difference or communication barrier. In the act of communication, clarity, truth, correctness, and appropriateness of the information was considered effective. Communication behaviours in an information system should be based on a well-structured communication path, considering the information symmetry of both parties [51]. Habermas believed that communication should be guided by mutual understanding, consensus should be reached between the two parties in the communication process and all participants in the system should be involved [52].

From the perspective of Habermas, nurses needed to face two aspects in the field of nursing. One was the responsibility they assumed for their service and the other was the obligation undertaken in operating the social system [53]. Mishler adopted Habermas’ point of view in the process of medical relationships in that doctor-patient communication was very difficult to control and understand. The researchers recorded the statements about doctor–patient communication in real life, analysed the linguistics and concluded that communication behaviour should be based on ethical definitions and that technology can realise the rationality of communication so that both sides can understand and communicate [54].

Our model demonstrated that communication mode is the core of the theoretical framework and the problems encountered in the study should be clarified to improve the communication mode. Using the Johari window model to analyse the known and unknown aspects of communication between the hospital and the patient can be improved in the applied theoretical framework and later, in the development process of the graphical health education tools (Table 5). The Johari window analysis method found that the problem of communication was composed of four dimensions in a matrix of 2 × 2 [55]: the process of long-term home care, the problem in each region, the improvement of communication behaviour and communication tools, and changing the method of communication between the hospital and the patient.

## 6. Conclusions

This study can assist designers to explore the theoretical basis of pictorial health education tools for nurses in the context of long-term care at home. The communication process is a complex process involving not only the home care operation details, interdisciplinary cooperation, and medical ethics, but attention also needs to be paid to the development of communication tools and morphology. Nurses need to acknowledge the long-term home care patient’s expression and the demands of home caregivers. They also need to cooperate with professional designers to develop pictorial health education tools and provide home caregivers with an easier method of understanding operational procedures and communication.

Interdisciplinary cooperation needs both groups to understand each other as much as possible without affecting each other’s operating conditions. This is difficult because cooperation needs both sides to have common knowledge and experiences, or at least respect for each other’s knowledge, experience, and operational methods. Cooperation in this study highlighted what both groups were good at; they had frequent question and answer sessions during the process, operational errors could be corrected and once the design professionals had developed the flowchart, it could be used for hospital nursing staff to teach home caregivers in the future.

## Figures and Tables

**Table 1:** The main scope and content of the interview.

Empty Frame	Interview Questions
Interdisciplinary cooperative norms [6]	1. Introduce research directions to seek cooperation and ask if the research can be conducted in the hospital. 2. What rules need to be adhered to?
The manner of nurses’ communication [34]	3. What are the basic conditions of the caregiver? 4. What cautions need to be taken when communicating with patients? 5. What are the basic communication methods between nurses and caregivers?
Problems in home care procedures [35]	6. What problems need to be solved in the process of home care? 7. What should the home caregivers do at home for intubated patients? 8. What methods does the nurse use to teach home caregivers skills?
Expectations for developing health education tools [36]	9. What specification of health education tools are more convenient to use? 10. From the nurse’s perspective, what are the requirements for the design of home care tools?
Nursing care for intubated patients [37]	11. What is the key content of nursing in the home care process? 12. What matters need attention in the home care process?

**Table 2:** Interview reliability.

Times	Researcher 1	Researcher 2	Researcher 3	Common Sentence	Mutual Agreement	Reliability
1	20	26	21	2	0.090	0.228
2	23	22	22	20	0.896	0.962

**Table 3:** Open coding and axial coding.

Axial Coding	The Theme	Open Coding	Role	Source
Method of interdisciplinary cooperation (AC01)	Researchers and nurses need to work together	*Researchers can follow them on the front line, the research topic may change, the data will be more refined.*	Hospital manager	CA01-51
	Help the nurse with real difficulties	*Learn as much as you can from nurses, observe their problems and help solve them. The design of the icon registration section has been developed and published before.*	Hospital manager	CA01-58
	Speaking right of cooperation	*So, let’s start with three-tube care, which is to give you the video and paperwork. Map out the steps and bring them up when we visit the home.*	Nurse	CB02-57
Medical research ethics (AC02)	Observe research ethics and avoid disputes	*When doing research, patients are involved and there will be patient privacy, so relevant regulations should be adhered to.*	Hospital manager	CA01-54
	Attitude of the patient and family	*We need the consent of the patient and the family first.*	Nurse	CB02-18
Communication methods (AC03)	Communication is important in nursing and details need to be improved	*(Researcher: During direct demonstration, in which cycle did the problem appear?) The nurse replied: Communication.*	Nurse	CB02-51
	How nurses teach and communicate with home caregivers	*There is no video, all procedures are on-the-spot demonstrations.*	Nurse	CB02-49
	The nurses explain the background of home caregivers	*Indonesia predominates, as does the Philippines.*	Nurse	CB02-26
	Existing communication tools are pen and paper	*Written on paper, Chinese characters.*	Nurse	CB02-83
	Nurses rely on personal experience to communicate and it is difficult for patients to express their true wishes	*Guess with experience and then describe the meaning roughly to family members. The patient only nods and shakes their head.*	Nurse	CB02-85
	There is a communication barrier between nurses and patients and it is very difficult for patients to express themselves, so nurses need to guess what patients think during communication.	*He (the patient) communicated with me using his hands and feet because he can only say a few words; the patient got angry. The patient can make breathing sounds, but I can probably understand them. In terms of communication, he only listens to what he wants to hear.*	Nurse	CB02-173
Health education tools form (AC04)	Nurses’ expectations of health education tools	*What if it’s A4 size? Wall charts are not convenient in case there is no place to hang them.*	Nurse	CB02-148
	Nurses use health education tools in the form of traditional printed products, but the size of the tools need to be easy to use	*(We) think you can use A4 size to make a small manual and then coil it into a book, it’s easy to turn pages. This will be easier than wall charts.*	Nurse	CB02-150
Health education tools development (AC05)	The development of health educational tools requires recording of specific processes, but some steps may not be completed in a simulated environment	*We can start with a medical dummy. But we need to know the structure of the medical dummy first. Whether the nasogastric tube can be inserted is uncertain.*	Nurse	CB02-37
	Uncertainty factors in the way health education tools are developed and it is important to find an appropriate way to demonstrate an operation	*We could look for a similar standard movie for your reference, or we could try to send you a video. It is mainly about the technology of placing tubes and care.*	Nurse	CB02-42
Intubation home care operation (AC06)	A step-by-step demonstration of the operating process is required	*That step will slowly decompose.*	Nurse	CB02-15
	Difficulties in the operation of home caregivers	*Most of the body cleaning is fine, only the technical aspects, such as turning the patient over and getting them in and out of bed.*	Nurse	CB02-65
	The main problems considered by nurses in home care is the aseptic operation of intubated patients	*In my opinion, intubation should not be the main focus of carers, but care should be mainly about disinfection and how to observe the principle of sterility.*	Nurse	CB02-156
	Operational considerations and concerns	*For example, matters needing attention in terms of the nasogastric tube as it is so long. We will measure and cut a suitable length and insert it. Then how to teach the family members and carers to confirm the tube is in and how to avoid it slipping.*	Nurse	CB02-159
	Nurses also realised that text might not be suitable for home care, so education tools were repeatedly proposed during operation	*You can’t read the text directly, but we can use a medical dummy. Then provide written information while giving guidance.*	Nurse	CB02-168

**Table 4:**
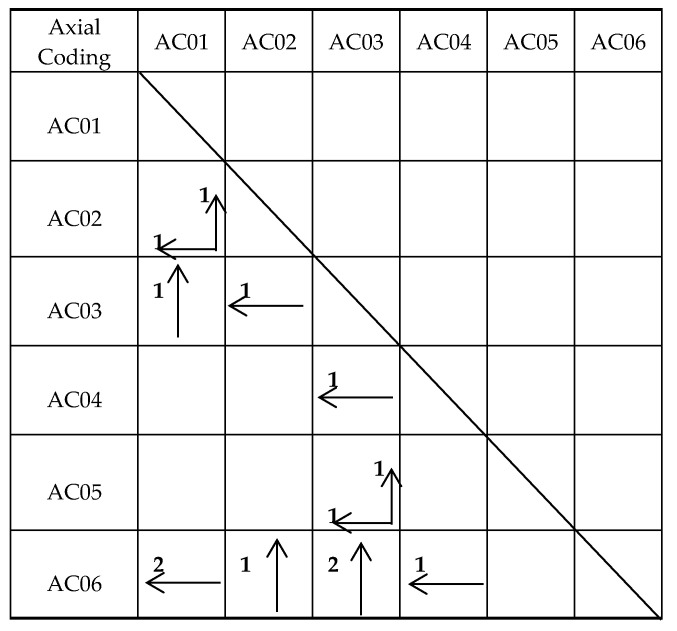
Selective coding table: Quantitative relationship between axial coding and interview facts.

**Table 5:** Johari Window analysis of communication between hospital staff and patients.

Intervention Tools: Communication Behaviours and Intervention of Communication Tools (Language, Text, Image, Body, etc.)		Hospital Staff: Doctors and Nurses	
		Known	Unknown
**Patient Side: Patients, family members and home caregivers**	**Known**	**Arena** ● The patient needs home care ● Existing symptoms of the patient ● The physical statement of the patient	**Blindspot** ● An accurate representation of a disease or symptom by the patient ● The influence of mood, tone, and attitude on the other communication side ● Communication in different situations
	**Unknown**	**Facade** ● Care methods ● Treatment of symptoms ● Methods of home care ● Steps of home care	**Unknown** ● The patient’s accuracy of expression ● Whether caregivers and family members have adequate health literacy ● Whether the patient’s side has an understanding of nursing knowledge
